# Analysis of Differences in the Degree of Biomechanical Adaptation according to Habituation to Different Heel Heights

**DOI:** 10.1155/2020/1854313

**Published:** 2020-06-01

**Authors:** Yu-Jin Cha

**Affiliations:** Department of Occupational Therapy, Semyung University, Jecheon 27136, Republic of Korea

## Abstract

This study aims to comprehensively investigate whether there are any differences in the degree of biomechanical adaptation according to habituation to different heel heights. The biomechanical characteristics of 54 subjects in 3 groups habituated to three heel heights (low, medium-high, and high heels) were evaluated by the measurement of surface EMG, myotonometer (e.g., measurement of muscle tension), foot pressure, and lumbosacral angle, and comparative analysis was carried out to find out whether they showed differences in the comfort visual analog scale (comfort VAS). Wearers of high-heeled shoes (6 cm or more in heel height), in foot pressure comparison, showed significantly high peak pressure in the mask of the hallux, high maximum peak EMG in the gastrocnemius medius (GM), and a high percentage of maximum voluntary isometric contraction (%MVIC) in the plantar flexor. Wearers of low-heeled shoes (3 cm and below in heel height) showed the highest plantar peak pressure in the lateral forefoot and midfoot, the highest contact area in midfoot, the highest %MVIC in the plantar flexion and dorsiflexion of the tibialis anterior (TA), and the highest stiffness in the TA, and they showed the lowest static balance ability with eyes open (EO) among the three groups. It was found that there were significant differences between those habituated to high-heeled shoes and those not habituated to high-heeled shoes and that longtime wearing of high-heeled shoes brings about biomechanical adaptive changes in the human body.

## 1. Introduction

High-heeled shoes have harmful effects on gait and lower extremity function and also cause changes in body alignment as they affect the center of gravity (COG) of the body [1]. It has been reported that they cause hallux valgus deformity, bring about changes in the lumbosacral angle, and also change pressure patterns leading to low back pain [2]. In addition, changes in kinetic and kinematic characteristics as well as biomechanical adaptation of the spine are required to absorb the increased vertical shock experienced while walking in high-heeled shoes [3].

Biomechanical changes caused by heel height also influence balance ability; the medial arch of the foot is raised, the body weight is shifted forward while walking, and there are changes in balance ability due to improper body alignment as well as the weakening of plantar flexion and muscular strength, along with changes in the muscle activation of the trunk and lower extremities [4]. Foot deformation or musculoskeletal abnormality leads to decreased body balance ability, which makes a normal gait difficult [5].

As a result of analyzing the changes of load in the foot according to heel heights, a conclusion was drawn that high heels increased the load on the forefoot, while releasing some load on the rearfoot, which could aggravate hallux valgus deformity [6]. There were significant differences between wearers of high-heeled shoes and those of low-heeled shoes, and the long-term wearing of high-heeled shoes resulted in differences such as lowered sensory function in the foot [7] and changes in the line of gravity for the ankle and knee joints (the biomechanical adaptation of the spine) [4]. Also, the wearers of high-heeled shoes showed significantly higher muscle activation of the biceps femoris and neurophysiological adaptation of the femoral muscles, whereas they felt less instability in the ankles than the wearers of low-heeled shoes [8].

The insole was designed and developed to prevent muscle fatigue by dispersing body weight and protect the ankle and knee joints through shock absorption in the functional aspect [9]. As a result, the insole is a conservative means that can provide comfort and stability to users, facilitate walking, and prevent and treat musculoskeletal disorders that may occur in the feet [10]. The total contact insole (TCI) reduced the heel pressure, decreased the impact force, redistributed the foot pressure, prevented the compensation strategy of the joint, and was particularly effective in the energy efficiency of the hip joint and correcting biomechanical alignment significantly more when it was applied to high-heeled shoes then it was applied to low-heeled shoes [11, 12].

Previous studies have investigated bodily changes occurring while wearing high-heeled shoes, but there are insufficient studies on body adaptation and biomechanical changes that may appear among those who wear high-heeled shoes for an extended period of time [8]. Wearers of low-heeled shoes showed differences such as lowered muscle activation of the rectus femoris and enhanced activation of the biceps femoris while standing in high-heeled shoes [13]. This data, however, is somewhat insufficient for a clear conclusion because only one kinetic aspect was investigated.

The purpose of this study is to comprehensively investigate whether there are any differences in the degree of biomechanical adaptation caused by habituation to different heel heights. For this, the measurements of surface EMG, myotonometer, foot pressure, and lumbosacral angle were carried out, and comparative analysis was performed to find out whether there are any differences in the comfort visual analog scale (comfort VAS) that can be attributed to changes in heel height.

## 2. Methods

### 2.1. Participants and Settings

The purpose and methods of this study were explained to the subjects in advance, and after informed consent to voluntarily participate in the research was obtained from women between 19 and 36, the experiments were conducted according to the procedure for the approval of the Institutional Review Board. There were a total of 54 subjects organized into three groups: a group of 18 women wearing high-heeled shoes 6 cm or more in heel height for 4 hours or more a day [14], a group of 17 women wearing medium high-heeled shoes 4-5 cm in heel height, and a group of 19 women wearing low-heeled shoes 3 cm or less in heel height. In other words, wearing high-heeled shoes (≥ 6 cm) was only applicable to Group A, not all subjects.

Those who had neurological or orthopedic problems, had experienced an orthopedic injury during the previous six months, had discomfort or complained of general pain throughout the body, had a deformed foot, or had psychiatric problems were excluded from the research. This study selected subjects of similar body indices who satisfied the WHO standard normal BMI of 18.5–24.9, in order to minimize the effects of stature, weight, foot size, etc. on experiment results and to prevent these factors from influencing balance performance ability. The period of research was from April 29 to May 17, 2019.

**Figure 1:**
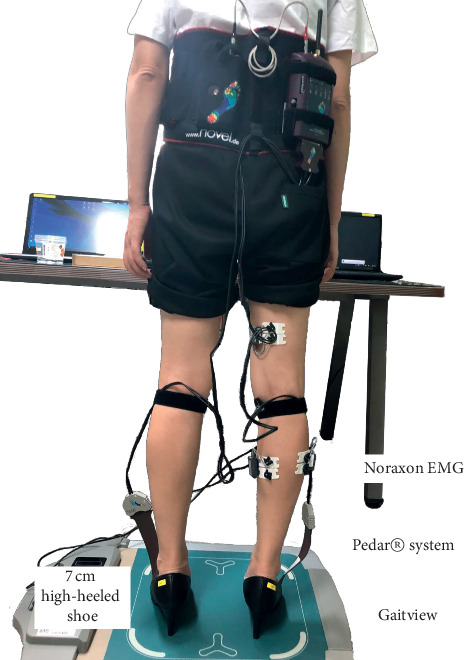
Experimental setup.

The high-heeled shoes used for the experiment were identical products 7 cm in heel height and 1 cm^2^ in heel area, and shoes in standard sizes of 230, 235, 240, 245, and 250 mm were prepared in accordance with the foot sizes of the subjects (Product Name: Bonita 45764). The heel height means the height difference between the front and the back of the shoe, and the heel area was determined by measuring the area of the heel touching the ground [15]. Measuring instruments and variables used in this study are as follows (Figure 1).

### 2.2. Data Collection

#### 2.2.1. In-Shoe Pressure Measuring System

**Figure 2:**
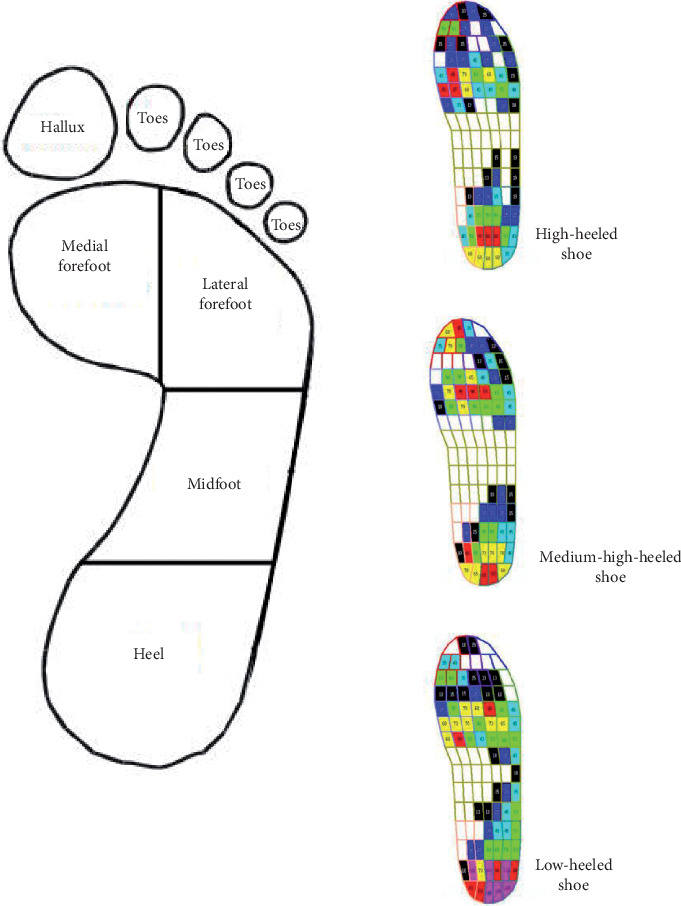
Six masks of the Pedar-X in-shoe pressure measuring system and the distribution of pressure across sole regions.

The plantar pressure distribution data was collected using the Pedar-X System (Novel Gmbh, Germany). The plantar pressure measuring system provides temporal and quantitative data about pressure on each part of the foot through a sensor attached to the insole and provides a wide range of data. It enables the analysis of the plantar center of pressure (COP) trajectory as well as peak force, peak pressure, impulse, duration of stance phase, and loading rate, making it very useful for diverse analysis [16]. To analyze the distribution of plantar pressure measured under experiment conditions, this study divided the foot into six anatomical regions (masks) (hallux, toes, medial forefoot, lateral forefoot, midfoot, and heel) and measured peak pressure (PP) and contact area (CA) (Figure 2).

#### 2.2.2. Surface EMG

**Figure 3:**
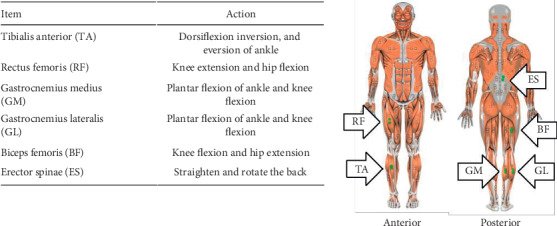
Surface electrodes attached points.

To measure muscle activation, the electromyograph of Noraxon EMG (Noraxon USA Inc., Scottsdale AZ, USA) and electromyogram software MR-XP Master Edition version 1.07 (Noraxon USA Inc., Scottsdale AZ, USA) linked to a personal computer to store the collected data were used. Root mean square (RMS) processing was applied after the full-wave rectification of EMG signals [17] (Figure 3).

Maximal voluntary isometric contraction (MVIC) was conducted in the position of the manual muscle test of the dorsiflexor and plantar flexor for the ankle of the dominant lower extremity (LE), as suggested by Hislop and Montgomery [18], maintained for a period of five seconds, and repeated and measured three times. To determine each subject's dominant leg, the subjects were asked to kick a ball, and the leg used for kicking the ball was determined to be the dominant leg [19].

#### 2.2.3. Myotonometer

**Figure 4:**
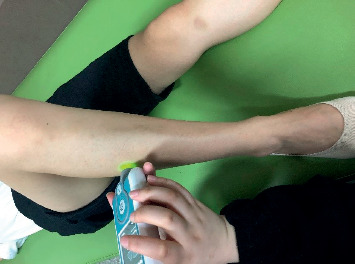
Muscle stiffness was measured using the MyotonPro device on muscle site.

The MyotonPRO device (MyotonPRO, Myoton Ltd., Estonia), a tactile soft-tissue measurement system, is a digital palpation device that enables the noninvasive assessment of mechanical properties, including tension, of soft tissues such as muscles and tendons [20–22]. And it has been reported that their measurements have high objectivity and reliability, without depending on testers, even among patients with Parkinson's disease or stroke as well as healthy adults [23]. The present study measured the subjects' muscular tension (*F*), stiffness (*S*), and elasticity (*D*) and selected four muscles to be measured, i.e., TA, RF, GM, and BF (Figure 4).

#### 2.2.4. Balance Assessment System (Gaitview System)

The Gaitview system can display on the monitor the COP and its moving distance while weight is loaded on the measuring plate, and colors and relative numerical values are displayed according to loading degree, while training feedback is possible by distributing weight applied to one leg. This study was performed using static balance tests where the subjects stand on both feet in shoes 7 cm high, with eyes open and closed. Three measurements were performed for 20 seconds per measurement, with a 10-second break between measurements, and then the average of the three measurements was calculated [4]. The Gaitview pro 1.01 analytics program was used, and the length of the path of the COP was measured.

#### 2.2.5. Functional Reach Test

**Figure 5:**
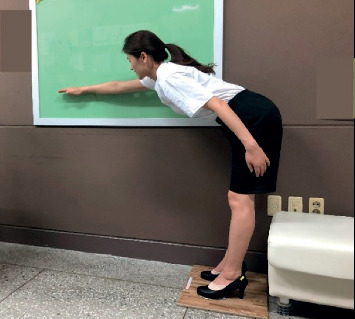
Functional reach test.

The functional reach test (FRT), which was developed by Duncan et al., is a validated test to measure dynamic balance ability. The FRT is a test that can measure the dynamic balance ability and flexibility while performing a functional task and it evaluates the limit of stability [24]. This study selected the FRT for measuring the dynamic balance ability because it is effective in predicting balance owing to its high validity and reliability and it can be easily applied in daily life without using expensive equipment. The subjects were asked to raise their arms to shoulder height in a standing position and reach as far forward as possible, and the maximal reach up to the end of the third finger was measured. In this study, the subjects in all three groups were asked to wear shoes for experiment 7 cm in heel height, the measurements of the FRT were conducted three times, and the average of the measurements was obtained (Figure 5).

#### 2.2.6. Pelvic ROM

The lumbosacral angle was measured using BROM (Back Range of Motion) II. The subject was asked to relax and directed to stand erect. With the posture of ordinary people as a reference, subjects were asked to place their feet 15 cm apart and to put their arms in a comfortable position. The distance between the two feet was marked on the floor for consistency, so the possible influence of changes in the distance between the feet on the lumbosacral angle in each condition could be excluded [17]. A one-minute break was put between the measurements.

#### 2.2.7. Intrarater Reliability

For intrarater reliability, the same rater obtained Cronbach's alpha for the results of the test-retest of the subjects' lumbosacral angle as measured by BROM II. It was found that ICC in the test-retest of BROM II was 0.939 and ICC for area 95% was 0.793, respectively [25]. In this study, Cronbach's alpha was obtained to investigate the intrarater reliability of the lumbosacral angle measured by BROM II, using the results of three measurements, excluding the maximum value and the minimum value, among five measurements for a total of 30 subjects, 10 each from Groups A, B, and C.

#### 2.2.8. Comfort Visual Analog Scale (Comfort VAS)

The comfort VAS was conducted to find a subjective comfort based on heel height and the duration that shoes were worn. The subjects were asked to specify the comfort levels of their own shoes and the shoes for the experiment by indicating positions along a continuous line between two endpoints. The left endpoint was 0, which means “very uncomfortable,” and the right endpoint was 10, which means “very comfortable.” A questionnaire survey of the subjects was conducted to measure the comfort level of the 7 cm heeled shoes used in the experiment [26, 27].

### 2.3. Research Procedure

To find out whether there are any differences in the degree of biomechanical adaptation (e.g., structure, function, and motion of the mechanical aspects of biological systems, using the methods of mechanics) according to habituation to different heel heights, the subjects were divided into three groups of wearers of high-heeled shoes (6 cm or more in heel height), wearers of medium high-heeled shoes (3–5 cm in heel heights), and wearers of low-heeled shoes (3 cm and below in heel heights). EMG electrodes were attached to the dominant leg of each subject. A Pedar® in-shoe pressure measuring system insole was placed on the sole of the standard shoes used in the experiment. The subject, wearing the 7 cm high standard shoes, stood on the measurement plate of the Gaitview system on both feet, three measurements were performed for 20 seconds per measurement, with a 10-second break between measurements, and then the average of the three measurements was calculated [4]. In this study, all subjects were standing on a plate while wearing high-heeled shoes (≥7 cm) because this study tried to find out if biomechanical adaptation varied depending on three different heel heights. In other words, this study aimed to validate the assumption that body adaptation and biomechanical changes would be different according to the height of commonly wearing shoes. After that, measurements were carried out in order for maximal voluntary isometric contraction (MVIC), functional reach test (FRT), myotonometer, and comfort VAS.

### 2.4. Statistical Analysis

The data for this study was processed and analyzed by using SPSS (version 22.0-Chicago, IL, USA). The mean and standard deviation of the subjects' general characteristics were obtained. For the analysis of homogeneity among the three groups, one-way ANOVA was performed. As for the differences in surface EMG, myotonometer, foot pressure, lumbosacral angle, and comfort visual analog scale (comfort VAS), one-way ANOVA was performed, and when it was judged that there were differences in the results between groups, a post hoc test (Dunnett T3) was performed. The effect size (ES) is known as Cohen's *d* [28], and the ES increases as the difference between the groups to be compared increases. To investigate intrarater reliability from the results of three measurements of the lumbar lordosis, Cronbach's alpha was obtained, using reliability analysis. The statistical significance level was *α* = 0.05.

## 3. Results

**Figure 6:**
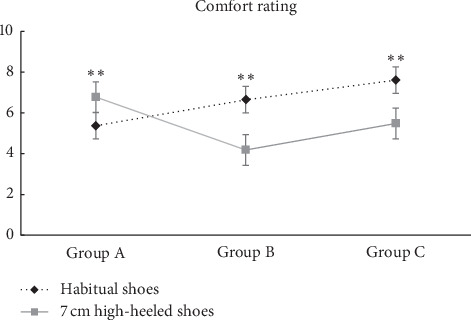
Comparison of comfort rating between 3 groups. ^*∗∗*^Significant difference between corresponding habitual shoes and 7 cm high-heeled shoes (*p* < 0.001).

The subjects of this study were 54 females in their 20 s, and their characteristics are as shown in Table 1. They were classified as those who had a past history of wearing high-heeled shoes 6 cm or more in heel height for 12 months or more (Group A), wearers of medium high-heeled shoes 4-5 cm in heel height (Group B), and wearers of low-heeled shoes 3 cm or below in heel height (Group C). As a result of homogeneity analysis among the three groups, it was found that there were no significant differences in age, weight, height, and shoes size among the groups, and so it may be said that they are homogeneous groups. On the other hand, they showed differences in comfort VAS between their habitual shoes and the 7 cm heeled shoes used for the experiment (*p* < 0.001). The group that answered that their habitual shoes were most comfortable was the wearers of low-heeled shoes (Group C), whereas the wearers of high-heeled shoes (Group A) indicated the highest level of comfort with the 7 cm heeled shoes used for the experiment (Figure 6).

### 3.1. Pressure Distribution

**Figure 7:**
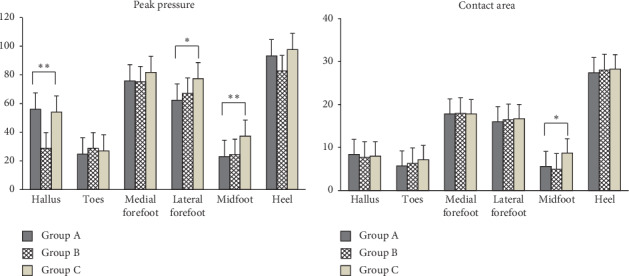
Comparison of mean pressure distribution by foot mask among the three groups.

With regard to peak pressure, in the mask of the hallux, it was significantly high among the wearers of high-heeled shoes (Group A) compared to Groups B and C, and in the mask of midfoot, it was significantly high among the wearers of low-heeled shoes (Group C) compared to Groups A and B. As for the contact area, in the mask of midfoot, it was significantly high among the wearers of low-heeled shoes (Group C) compared to Groups A and B (*p* < 0.05) (*p* < 0.001) (Table 2) (Figure 7).

### 3.2. Surface EMG

The degree of EMG activity (RMS EMG) showed significant differences in GM, GL, and ES among the three groups; in GL and GS, it was the highest among wearers of medium-heeled shoes (Group B), followed by Groups C and A. The maximum peak EMG showed significant differences in RF, GM, BF, and ES; in RF and BF, it was the highest in the order of C, A, and B; in GM, in the order of A, C, and B; and in ES, in the order of B, C, and A, respectively (*p* < 0.05) (*p* < 0.001) (Table 3).

### 3.3. Myotonometer

The groups showed significant differences in the muscle tone (*F*) of TA, RF, and GM, the muscle stiffness (*S*) of TA, and elasticity (*D*) of BF. The muscle tone of RF and GM was the highest among the wearers of high-heeled shoes (Group A), followed by Group C and Group B. The muscle tone (*F*) of TA was the highest among the wearers of low-heeled shoes (Group C), followed by Group A and Group B. The stiffness (*S*) of TA was the highest among the wearers of low-heeled shoes (Group C), followed by Group A and Group B. The elasticity of BF was the highest among the wearers of medium high-heeled shoes (Group B), followed by Group C and Group A (*p* < 0.05) (*p* < 0.001) (Table 4).

### 3.4. Balance

The groups showed significant differences in static balance ability with open eyes, and the static balance ability was the highest among the wearers of low-heeled shoes (Group C), followed by Group B and Group A. It was the wearers of high-heeled shoes (Group A) who showed the most stable balance ability after putting on the 7 cm shoes for the experiment (*p* < 0.05) (Table 5).

### 3.5. Pelvic ROM

#### 3.5.1. Intrarater Reliability

As for intrarater reliability, the same rater obtained Cronbach's alpha for the results of the test-retest of the subjects' lumbosacral angle measured by BROM II. Cronbach's alpha for Groups A, B, and C was found to be 0.987, 0.906, and 0.996, respectively. In the case of Group B, the intrarater reliability showed 0.906, lower than the other groups; however, all three groups showed relatively high intrarater reliability. The groups showed significant differences in the lumbosacral angle, and the lumbosacral angle was the highest among the wearers of high-heeled shoes (Group A), followed by Group B and Group C. That is, it was Group A that showed the largest lumbosacral angle (*p* < 0.05) (Table 6).

## 4. Discussion

High-heeled shoes are considered one factor related to musculoskeletal problems involving knees and legs because they can cause changes in the static condition and dynamic movement of the body. Therefore, this study was undertaken with the intent of comprehensively investigating whether there are any differences in the degree of biomechanical adaptation caused by habitation to different heel heights.

As a result of this study, it was found that, in the comparison of plantar pressure, while wearers of high-heeled shoes showed significantly high peak pressure in the mask of the hallux, wearers of low-heeled shoes showed significantly high peak pressure in the masks of lateral forefoot and midfoot. And wearers of low-heeled shoes showed a significantly high contact area in the mask of midfoot. It is thought that this is due to the fact that wearers of high-heeled shoes are habituated to a COG adapted to a forward shift. This supports the findings of previous studies that high-heeled shoes raise pressure on the forefoot [17, 29] and increase shock force, pressure on the internal forefoot, and discomfort [11].

In the comparison of EMG activation, the wearers of medium high-heeled shoes showed the highest muscle activation in GL and ES; the wearers of low-heeled shoes showed the highest maximum peak EMG in RF and BF, the wearers of high-heeled shoes in GM, and the wearers of medium high-heeled shoes in ES, respectively. In the comparison of the %MVIC, the wearers of low-heeled shoes showed the highest %MVIC in the plantar flexion and dorsiflexion of TA and the wearers of medium-heeled shoes in the plantar flexion of GL, respectively. These findings are consistent with the findings of previous studies showing that habituation to high-heeled shoes resulted in neurophysiological adaptation in the rectus femoris (RF) and biceps femoris (BF) [8, 30, 31] and increased the muscle activation of plantar flexor [32]. They were also consistent with the findings of a study showing that when males walked while wearing high-heeled shoes, the maximal muscle activation of their gastrocnemius decreased [33].

In comparison using the myotonometer, the muscle tone of RF and GM was the highest among the wearers of high-heeled shoes, and the muscle tone (F) of TA was the highest among the wearers of low-heeled shoes. Stiffness (S) of TA was the highest among the wearers of low-heeled shoes, and the elasticity of BF was the highest among the wearers of medium high-heeled shoes. This supported the findings of previous studies showing that high-heeled shoes increased the muscle activation of plantar flexor, inducing muscular imbalance, and caused musculoskeletal problems such as disc issues, degenerative arthritis, swollen and numb feet, and changes in body shape [30, 32].

Finally, wearing high-heeled shoes for a long time causes changes in the position of body segments and the center of gravity, which results in compensatory kinematic and kinetic changes [14]. High heels increase pelvic inclination due to the compensation of postural alignment control mechanisms due to the biomechanical adaptive changes of the spine and lower extremity joints and induce muscular fatigue, the decrease of balance ability, and an increase in the risk of falling [34]. And it has been reported that high-heeled shoes change spinal curvature according to the size of the lumbosacral angle and increase the shearing force to cause structural changes that put pressure on posterior ligament and facet joint, thereby becoming a factor in causing lower back pain [35].

Differences in heel height also cause changes in balance ability. A study showing significant changes in static balance ability suggests that balance ability was most stable among the wearers of 3 cm heels [36], followed by barefoot and 7 cm heels, which is different from the findings of this study. It is difficult to compare the findings because the subjects and the heel heights of shoes used for the experiments were different. Another study shows no significant difference in static balance ability according to heel heights both with eyes open and with eyes closed, but its findings are consistent with the findings of this study in that the most stable balance ability was found when the heel height was 7 cm in both groups [4]. At this time, good balance ability was found overall among women habituated to high heels, but significant differences in static balance ability were found according to whether they were habituated to high heels or not [37]. This seems to be because women habituated to high-heeled shoes showed physiological adaptation [33].

The greatest change in the lumbosacral angle in standing position was in those who wore high-heeled shoes. This result is consistent with the findings of previous studies suggesting that the higher the heel height, the greater the muscle activation of the lower extremities and the lumbosacral angle, and this shows that the increase in heel height acts as a factor that increases the muscle tone of a particular region and induces fatigue [8]. Particularly, increased load on the lumbar vertebra is involved in the formation of posture during walking and the movement of the central point of the human body and thus has effects on the stability of the human body and influences muscle activation of every region accordingly. Wearing high-heeled shoes increases the plantar flexion of the ankle joints and brings about changes in the relative position of bones within the joint and the muscle origin [37].

It was found that there were significant differences between those habituated to high-heeled shoes and those not habituated to high-heeled shoes and that longtime wearing of high-heeled shoes brings about biomechanical adaptive changes in the human body. It is expected that foot pressure, surface EMG, or proprioception will be different between the two conditions (eyes open and eyes close). However, this study conducted the test only under the eyes open condition because people live their lives and walk around with their eyes open. Therefore, it is believed that future studies need to compare the two conditions (eyes open and eyes close). A secondary limitation of this study is that the subjects were mostly those in their early twenties, and thus it is difficult to generalize the findings across ages. And it is deemed that future studies will need to conduct research on adult women of various age brackets.

Further studies will have to be conducted continuously to link various changes in the sensory system to kinematic analysis regarding the movement of COG and the efficiency of movement according to habituation to heel heights. In addition, to prevent disorders caused by habituation to heel heights, research should be continued regarding the materials and structure of insoles that can disperse pressure evenly in consideration of differentiated shock variables.

## 5. Conclusion

This study was conducted to investigate whether there are any differences in the degree of biomechanical adaptation caused by habitation to different heel heights. For this, the subjects' biomechanical characteristics were evaluated by the measurement of surface EMG, myotonometer, foot pressure, and lumbosacral angle, and comparative analysis was carried out comprehensively to find out whether the subjects showed differences in the comfort VAS. The wearers of high-heeled shoes (Group A) showed significantly high peak pressure in the masks of hallux and midfoot, a significantly high contact area in the mask of midfoot, high maximum peak EMG in GM, and more active muscle activation in plantar flexor. In addition, they showed the highest muscle tone of RF and GM, the largest lumbosacral angle, and the most stable static balance ability (COP) in 7 cm heels with eyes open.

The wearers of medium-heeled shoes (Group B) showed the highest muscle activation and maximum peak EMG in ES, the highest %MVIC in the plantar flexion, and the highest elasticity in BF. The wearers of low-heeled shoes (Group C) showed the highest plantar peak pressure in the mask of the lateral forefoot, the highest %MVIC in the plantar flexion and dorsiflexion of TA, and the highest stiffness in TA.

In conclusion, it was found that there were significant differences between those habituated to high-heeled shoes (Group A) and those not habituated to high-heeled shoes (Groups B and C) and that wearing of high-heeled shoes brings about biomechanical adaptive changes in the human body. On the basis of these findings, this study intends to provide basic data for developing customized insoles that have aesthetic factors, convenience, and biocompatibility and are also able to disperse pressure and absorb and release shocks from high-heeled shoes differentiated according to habituation by heel height.

## Figures and Tables

**Table 1:** General characteristics of subjects (*N* = 54).

		Group A^a^ (*N* = 19)	Group B^b^ (*N* = 17)	Group C^c^ (*N* = 18)	*F*	*p* ^§^
Age (years)		20.11 ± 1.59^†^	20.11 ± 1.59	19.66 ± 1.14	2.731	0.075
Weight (kg)		53.29 ± 4.06	55.00 ± 3.35	54.11 ± 5.09	1.216	0.305
Height (cm)		167.45 ± 3.13	166.02 ± 2.96	165.78 ± 3.87	1.353	0.268
Shoes size (mm)		240 ± 3.22	244.34 ± 2.21	246.23 ± 2.56	10.692	0.094
Comfort rating	Habitual shoes	5.37 ± 1.46	6.65 ± 1.58	7.61 ± 2.20	7.448	0.001^*∗∗*^
	7 cm high-heeled shoe	6.78 ± 2.02	4.18 ± 1.78	5.48 ± 2.18	7.863	0.001^*∗∗*^

^*∗*^ *p* < 0.05 and ^*∗∗*^*p* < 0.001. ^†^Mean ± SD. ^§^Statistical differences are the differences between all three groups. ^a^Over 12 months, 4 times a week, and over 6 cm heeled shoes group. ^b^Over 12 months, 4 times a week, and 4-5 cm heeled shoes group. ^c^Over 12 months, 4 times a week, and below 3 cm heeled shoes group.

**Table 2:** Comparison of mean pressure distribution by mask according to habituation to different heel heights.

Variables		Group A^a^	Group B^b^	Group C^c^	*F*/*p*	ES^§^	Post hoc
Hallux	PP (kPa)	56.04 ± 13.13^†^	28.85 ± 14.20	54.04 ± 19.58	15.960/0.000^*∗∗*^	3.09	A > C > B (Dunnett T3)
	CA (cm^2^)	8.39 ± 1.52	7.71 ± 0.71	8.00 ± 1.77	1.035/0.363	0.24	
Toes	PP (kPa)	24.78 ± 7.93	28.85 ± 14.20	26.94 ± 7.10	0.730/0.487	0.53	
	CA (cm^2^)	5.71 ± 1.93	6.27 ± 2.83	7.16 ± 2.09	1.903/0.160	0.40	
Medial forefoot	PP (kPa)	75.77 ± 20.30	75.09 ± 24.88	81.75 ± 15.96	0.567/0.571	0.66	
	CA (cm^2^)	17.79 ± 1.73	17.93 ± 2.45	17.82 ± 2.27	0.037/0.964	0.04	
Lateral forefoot	PP (kPa)	62.35 ± 13.12	67.05 ± 18.75	77.39 ± 15.14	4.385/0.017^*∗*^	2.11	C > B > A (Dunnett T3)
	CA (cm^2^)	16.01 ± 1.90	16.47 ± 1.80	16.69 ± 1.92	0.621/0.541	0.21	
Midfoot	PP (kPa)	23.03 ± 9.36	24.29 ± 5.46	37.22 ± 14.00	10.580/0.000^*∗∗*^	2.07	C > B > A (Dunnett T3)
	CA (cm^2^)	5.60 ± 3.57	4.93 ± 3.78	8.70 ± 3.62	5.527/0.007^*∗*^	0.85	C > A > B (Dunnett T3)
Heel	PP (kPa)	93.24 ± 18.11	82.75 ± 20.70	97.69 ± 17.94	2.862/0.066	1.42	
	CA (cm^2^)	27.43 ± 1.52	28.05 ± 0.44	28.26 ± 2.58	0.972/0.385	0.29	

^*∗*^ *p* < 0.05 and ^*∗∗*^*p* < 0.001. ^†^Mean ± SD. ^§^Effect size. ^a^Over 12 months, 4 times a week, and over 6 cm heeled shoes group. ^b^Over 12 months, 4 times a week, and 4-5 cm heeled shoes group. ^c^Over 12 months, 4 times a week, and below 3 cm heeled shoes group. CA, contact area; PP, peak pressure.

**Table 3:** Comparison of muscle activation according to habituation to different heel heights.

Variables			Group A^a^	Group B^b^	Group C^c^	*F*/*p*	ES^§^	Post hoc
Muscle activation	TA	RMS EMG (*μ*V)	6.07 ± 3.22^†^	8.20 ± 4.76	7.04 ± 3.23	1.431/0.249	0.45	
		Maximum peak EMG (*μ*V)	329.84 ± 146.42	384.50 ± 189.32	339.76 ± 169.95	0.523/0.596	1.81	
	RF	RMS EMG (*μ*V)	8.10 ± 7.02	4.31 ± 2.89	6.87 ± 5.65	2.163/0.1260.69	0.69	
		Maximum peak EMG (*μ*V)	260.80 ± 182.39	144.28 ± 61.45	274.99 ± 189.85	3.583/0.035^*∗*^	4.79	C > A > B (Dunnett T3)
	GM	RMS EMG (*μ*V)	9.96 ± 3.21	14.98 ± 11.64	7.19 ± 4.99	4.824/0.012^*∗*^	1.23	B > A > C (Dunnett T3)
		Maximum peak EMG (*μ*V)	487.79 ± 194.26	197.95 ± 43.08	324.94 ± 130.18	19.424/0.000^*∗∗*^	10.73	A > C > B (Dunnett T3)
	GL	RMS EMG (*μ*V)	5.66 ± 3.97	9.01 ± 4.23	6.38 ± 2.82	3.925/0.026^*∗*^	0.74	B > C > A (Dunnett T3)
		Maximum peak EMG (*μ*V)	286.89 ± 207.10	406.34 ± 233.44	327.06 ± 141.82	1.667/0.199	3.54	
	BF	RMS EMG (*μ*V)	2.69 ± 1.74	4.92 ± 6.95	3.70 ± 2.24	1.234/0.300	0.48	
		Maximum peak EMG (*μ*V)	148.56 ± 88.33	108.28 ± 31.39	202.81 ± 100.61	6.014/0.005^*∗*^	4.46	C > A > B (Dunnett T3)
	ES	RMS EMG (*μ*V)	2.98 ± 0.73	5.89 ± 2.99	3.54 ± 2.00	9.617/0.000^*∗∗*^	0.90	B > C > A (Dunnett T3)
		Maximum peak EMG (*μ*V)	156.26 ± 38.95	288.26 ± 158.82	193.01 ± 92.77	7.206/0.002	5.60	B > C > A (Dunnett T3)

%MVIC	TA	Plantar flexion	2.98 ± 1.27^†^	2.94 ± 0.35	4.39 ± 2.93	3.507/0.038^*∗*^	0.55	C > A > B (Dunnett T3)
		Dorsiflexion	0.56 ± 0.48	0.82 ± 0.65	0.96 ± 5.20	1.450/0.003^*∗*^	0.12	C > B > A (Dunnett T3)
	GM	Plantar flexion	12.57 ± 10.49	4.15 ± 2.62	6.92 ± 4.80	6.620/0.310	1.44	
		Dorsiflexion	2.90 ± 2.11	5.90 ± 4.50	4.06 ± 0.99	3.654/0.244	0.77	
	GL	Plantar flexion	2.86 ± 1.18	4.39 ± 3.15	4.27 ± 3.09	1.198/0.033^*∗*^	0.45	B > C > A (Dunnett T3)
		Dorsiflexion	6.52 ± 4.63	8.07 ± 4.89	7.16 ± 4.90	0.471/0.627	0.29	

^*∗*^ *p* < 0.05 and ^*∗∗*^*p* < 0.001. ^†^Mean ± SD. ^§^Effect size. ^a^Over 12 months, 4 times a week, and over 6 cm heeled shoes group. ^b^Over 12 months, 4 times a week, and 4-5 cm heeled shoes group. ^c^Over 12 months, 4 times a week, and below 3 cm heeled shoes group. TA, tibialis anterior; RF, rectus femoris; GM, medial gastrocnemius; GL, lateral gastrocnemius; BF, biceps femoris; ES, thoracic erector spinae.

**Table 4:** Comparison of muscle tone (*F*), stiffness (*S*), and elasticity (*D*) according to habituation to different heel heights.

Variables		Group A^a^	Group B^b^	Group C^c^	*F*/*p*	ES^§^	Post hoc
TA	*F* (Hz)	19.07 ± 1.00^†^	17.52 ± 1.44	19.34 ± 1.99	7.209/0.002^*∗*^	0.65	C > A > B (Dunnett T3)
	*S* (N/m)	385.26 ± 24.56	367.29 ± 55.48	411.83 ± 67.11	3.292/.045^*∗*^	2.58	C > A > B (Dunnett T3)
	*D*	1.24 ± 0.17	1.23 ± 0.23	1.15 ± 0.19	1.186/0.314	0.09	
RF	*F* (Hz)	14.52 ± 1.13	13.35 ± 0.44	14.13 ± 0.91	7.982/0.001^*∗∗*^	0.52	A > C > B (Dunnett T3)
	*S* (N/m)	232.95 ± 30.94	216.76 ± 11.80	226.78 ± 23.91	2.076/0.136	1.41	
	*D*	1.20 ± 0.22	1.28 ± 0.14	1.20 ± 0.26	0.832/0.441	0.08	
GM	*F* (Hz)	16.15 ± 1.67	14.71 ± 1.50	15.37 ± 1.54	3.756/0.030^*∗*^	0.47	A > C > B (Dunnett T3)
	*S* (N/m)	262.79 ± 35.64	238.82 ± 33.46	262.50 ± 31.17	2.943/0.062	1.91	
	*D*	1.40 ± 0.21	1.48 ± 0.09	1.40 ± 0.15	1.390/0.258	0.10	
BF	*F* (Hz)	13.82 ± 1.77	13.60 ± 1.22	14.40 ± 0.87	1.672/0.198	0.30	
	*S* (N/m)	207.32 ± 41.09	227.47 ± 26.41	221.06 ± 23.36	1.936/0.155	1.54	
	*D*	1.03 ± 0.15	1.22 ± 0.08	1.08 ± 0.23	6.086/0.004^*∗*^	0.20	B > C > A (Dunnett T3)

^*∗*^ *p* < 0.05 and ^*∗∗*^*p* < 0.001. ^†^Mean ± SD. ^§^Effect size. ^a^Over 12 months, 4 times a week, and over 6 cm heeled shoes group. ^b^Over 12 months, 4 times a week, and 4-5 cm heeled shoes group. ^c^Over 12 months, 4 times a week, and below 3 cm heeled shoes group. TA, tibialis anterior; RF, rectus femoris; GM, medial gastrocnemius; BF, biceps femoris.

**Table 5:** Comparison of balance ability according to habituation to different heel heights.

		Group A^a^	Group B^b^	Group C^c^	*F*/*p*	ES^§^	Post hoc
Static balance (COP)	EO	103.67 ± 13.15^†^	133.42 ± 64.66	162.47 ± 96.81	3.578/0.035^*∗*^	3.19	C > B > A (Dunnett T3)
	EC	141.74 ± 44.07	120.23 ± 41.69	157.79 ± 80.88	1.828/0.171	2.70	
FRT		31.14 ± 8.52	32.04 ± 6.65	27.42 ± 6.02	2.080/0.136	0.75	

^*∗*^ *p* < 0.05. ^†^Mean ± SD. ^§^Effect size. ^a^Over 12 months, 4 times a week, and over 6 cm heeled shoes group. ^b^Over 12 months, 4 times a week, and 4-5 cm heeled shoes group. ^c^Over 12 months, 4 times a week, and below 3 cm heeled shoes group. EO, eye opening; EC, eye close; COP, center of pressure; FRT, functional reach test.

**Table 6:** Difference in lumbosacral angle and Cronbach *α* according to habituation to different heel heights.

	Group A^a^	Group B^b^	Group C^c^	*F*/*p*	ES^§^	Post hoc
Pelvic ROM (°)	94.02 ± 8.80^†^	91.56 ± 7.32	85.69 ± 11.66	3.756/0.030^*∗*^	1.16	*A* > *B* > C (Dunnett T3)
Cronbach *α*	0.987^*∗∗*^	0.906^*∗∗*^	0.996^*∗∗*^			

^*∗*^ *p* < 0.05. ^†^Mean ± SD. ^§^Effect size. ^a^Over 12 months, 4 times a week, and over 6 cm heeled shoes group. ^b^Over 12 months, 4 times a week, and 4-5 cm heeled shoes group. ^c^Over 12 months, 4 times a week, and below 3 cm heeled shoes group.

## Data Availability

The data used to support the findings of this study are available from the author upon request.
